# Association of AXIN2 s2240308 C>T, rs1133683 C>T, rs7224837 A>G Polymorphisms with Susceptibility to Breast Cancer

**DOI:** 10.31557/APJCP.2021.22.8.2717

**Published:** 2021-08

**Authors:** Soheila Sayad, Mahdieh Abdi-Gamsae, Jamal Jafari-Nedooshan, Meraj Farbod, Seyed Alireza Dastgheib, Mojgan Karimi-Zarchi, Fatemeh Asadian, Hossein Neamatzadeh

**Affiliations:** 1 *Department of Surgery, Tehran University of Medical Sciences, Tehran, Iran. *; 2 *Department of Biology, Shahr-e-Qods Branch, Islamic Azad University, Tehran, Iran.*; 3 *Department of Surgery, Shahid Sadoughi University of Medical Sciences, Yazd, Iran. *; 4 *Cancer Institute, Imam Khomeini Hospital, Tehran University of Medical Sciences, Tehran, Iran.*; 5 *Department of Medical Genetics, School of Medicine, Shiraz University of Medical Sciences, Shiraz, Iran. *; 6 *Department of Obstetrics and Gynecology, Iran University of Medical Sciences, Tehran, Iran. *; 7 *Endometriosis Research Center, Iran University of Medical Sciences, Tehran, Iran. *; 8 *Department of Medical Laboratory Sciences, School of Paramedical Sciences, Shiraz University of Medical Sciences, Shiraz, Iran. *; 9 *Department of Medical Genetics, Shahid Sadoughi University of Medical Sciences, Yazd, Iran. *; 10 *Mother and Newborn Health Research Center, Shahid Sadoughi University of Medical Sciences, Yazd, Iran. *

**Keywords:** Breast cancer, AXIN2, polymorphisms- association, susceptibility

## Abstract

**Background:**

The association of genetic polymorphisms at Axis inhibition protein 2 (*AXIN2*) gene and susceptibility to different cancer has attracted much interest. The present study aimed to evaluate the association between AXIN2 rs2240308 C>T, rs1133683 C>T, rs7224837 A>G polymorphisms with susceptibility to breast cancer.

**Methods:**

A polymerase chain reaction-restriction fragment length polymorphism (PCR-RFLP) assay was designed to genotype the AXIN2 rs2240308 C>T, rs1133683 C>T, rs7224837 A>G polymorphisms among 150 breast cancer patients and 150 healthy subjects.

**Results:**

The frequencies of these genetic variants were in agreement with Hardy-Weinberg equilibrium in healthy controls (p>0.05). The frequencies of AXIN2 rs2240308 C>T, rs1133683 C>T, rs7224837 A>G genotypes were similar in breast cancer patients and controls. There was no a significant association between the AXIN2 SNP and risk of breast cancer.

**Conclusion:**

The impact of AXIN2 polymorphisms in the breast cancer development remains unclear. Our results indicated that AXIN2 rs2240308, rs7224837 and rs1133683 polymorphisms did not contribute to increased risk of breast cancer. More studies with larger sample sizes and diverse ethnicities are warranted to verify our finding.

## Introduction

Breast cancer is the most common type of epithelial cancer among women globally, with an average risk of 12% to develop in normal women life (Tavakoli-Koopaei et al., 2020; Najminejad et al., 2020; Ghorbani et al., 2021). Breast cancer has also the highest incidence rate and the second death rates among all types of cancer (Motamedi et al., 2012; Sung et al., 2021). Breast cancer is a multifactorial, polygenic and heterogeneous disease which aggressive subtypes are characterized by faster growth rates, increased capability to invade and metastasize, leading to poorer clinical outcomes (Bianchini et al., 2016; Feng et al., 2018; Gooding and Schiemann, 2020). The incidence of breast cancer has been increasing in the world where it accounts for 6.9% of all cancers and 684,996 deaths in 2020 (Esmaeili et al., 2021; Sung et al., 2021). However, worldwide differences in incidence and mortality of this disease are largely explained by age and country-level income (Bellanger et al., 2018). It is evident that the increasing trend in incidence is mainly attributed to reproductive and lifestyle patterns such as older age at first birth, decrease in childbearing and breastfeeding, lower physical activity, and obesity (Bellanger et al., 2018; Sheikhpour et al., 2018; Moghimi et al., 2018; Francies et al., 2020). Breast cancer is a complex disease involving hereditary and environmental risk factors. A genetic (inherited) component in breast cancer is well established in the etiology of breast cancer (Kamali et al., 2015; Moghimi et al., 2018). Estimation of heritability based on twin studies found 25 to 31% to be explained by genetic factors (Möller et al., 2016). High-risk women are primarily identified on the basis of family history and mutation screening of the *BRCA1* and* BRCA2* genes, which responsible for a lifetime risk between 50 and 85% and account for approximately 15% of familial breast cancer cases (Forat-Yazdi et al., 2015; Neamatzadeh et al., 2015).

To date, a large effort to investigate the genetic component of breast cancer has been performed (De Medina et al., 2010; Li et al., 2021). The Wnt signaling pathway plays a crucial role during embryogenesis, while aberrations in this pathway are implicated in a variety of human cancers (Gajos-Michniewicz and Czyz, 2020; Guan et al., 2020). Axis inhibition protein 2 (AXIN2) also known as axin-like protein (Axil), a key component of the Wnt signaling pathway, plays an important role in the regulation of cell proliferation, cytometaplasia, migration, apoptosis and other important cellular functions, and it has demonstrated a close relationship with the development of some cancers (Xu et al., 2020). AXIN2 is a scaffold protein which is required for the phosphorylation of β-catenin. Stabilization of AXIN2 protein in cancer cell lines inhibited Wnt signaling, migration, and under low serum conditions, reduced colony formation (Koni et al., 2020; Liu et al., 2021). In addition to the many AXIN2 mutations reported in various cancer types, a few reports have linked AXIN2 SNPs to the disease risk in breast cancer. AXIN2 mutations have also been associated with the development of oral clefts, such as cleft lip and cleft palate (Noroozi et al., 2020).

The *AXIN2* gene is located on chromosome 17q23-q24, which belongs to a heterozygosity region that frequently loss in different cancers in human (Gong et al., 2015; Yu et al., 2017; Li et al., 2021). It has been suggested that polymorphisms at *AXIN2* gene are associated with the risk of developing of breast cancer. Moreover, it can be targeted by miR143HG/miR-1275 to regulate breast cancer progression by modulating the Wnt/β-catenin pathway (Li et al., 2021). Although numerous genetic association studies on breast cancer have been published, there are few a few studies about association of *AXIN2 *gene with breast cancer risk (Ren et al., 2019). To date, several number polymorphisms have been identified at AXIN2 region, including rs2240308 (exon1), rs9915936 (exon5), rs1133683 (exon5), and rs4072245 (intron7), which some of them most frequently studied in relation to risk of cancer. Here, the authors aimed to evaluate the association between AXIN2 rs2240308 C>T, rs1133683 C>T, rs7224837 A>G polymorphisms with susceptibility to breast cancer.

## Materials and Methods

*Subjects and Basic Characteristics*

All procedures in this study were carried out in accordance with the ethical standards of the institutional or national research committee and with the 1964 Declaration of Helsinki and its later amendments or comparable ethical standards. The objective of the study was fully explained to all participants and a written informed consent was obtained from each participants. This study was approved by the Ethics Committee of the Azad University. This study included 150 diagnosed with breast cancer and 150 healthy subjects without history of cancer.

*DNA extraction*

Genomic DNA was extracted from 200 μL of peripheral blood of all subjects with EDTA as an anticoagulant. A Commercially available kit for DNA extraction was used for DNA extraction, according to the manufacturer’s instructions kit (purchased from GeneAll Co., LTD). AXIN2 rs2240308, rs7224837 and rs1133683 polymorphisms were genotyped by polymerase chain reaction-restriction fragment length polymorphism (PCR-RFLP) using previously reported primers (Table 1). PCR was performed in a reaction volume of 25 µl containing 100 ng of genomic DNA. The PCR mixtures consisted of 10 pmol of the appropriate amplification primers, 5 nmol each of 4 deoxynucleotide triphosphates (Fermentas), 2.5 Units of Taq DNA polymerase (Fermentas), 10 mmol/l Tris-HCl (pH 8.3 at 25˚C), 50 mmol/l KCl and 1.5 mmol/l MgCl2. PCR conditions consisted of an initial denaturation at 94˚C for 5 min followed by 30 cycles of 94˚C for 30 sec, 60˚C for 30 sec and 72˚C for 60 sec, and lastly 1 cycle at 72˚C for 5 min. Then, PCR products were digested with 5 Units of appropriate restriction enzymes (Table 1) in a total reaction volume of 10 µl containing 1X reaction buffer for 5 h at 37˚C temperature. Following digestion, DNA fragments were separated on 2.5% agarose gels. Table 1 shows the digested DNA fragment sizes.

*Statistical analysis*

The chi-square test or Fisher’s exact test was performed to examine the differences between cases and controls in terms of mean age and gender. Hardy-Weinberg equilibrium (HWE) was used for the distributions of AXIN2 rs2240308, rs7224837 and rs1133683 polymorphisms in healthy subjects performed by the chi square (*χ*^2^) test. The associations of AXIN2 rs2240308, rs7224837 and rs1133683 polymorphisms with breast cancer risk was calculated by using the odds ratio (OR) and 95% confidence interval (CI) (Bahrami et al., 2020; Jarahzadeh et al., 2021). Statistical analyses were carried out using SPSS version 19.0 (SPSS Co., Chicago, IL, USA) for Windows.

## Results

Table 2 shows the main results of AXIN2 rs2240308, rs7224837 and rs1133683 polymorphisms genotyping. P-value of Hardy-Weinberg equilibrium test for AXIN2 s2240308 C>T, rs1133683 C>T, rs7224837 A>G polymorphisms in healthy subjects was 0.632, 0.197 and 0.545, respectively. The rs2240308 C>T, CC, CT and TT genotype frequencies were 48.0%, 42.7% and 9.3% in breast cancer patients versus 56.0%, 36.7% and 7.3% in controls, respectively. The frequency of minor allele was 30.6% in n breast cancer cases and 25.7% in healthy subjects. The rs1133683 C>T, CC, CT and TT genotype frequencies were 30.7%, 51.3%, and 18.0% in breast cancer cases versus 32.7%, 53.3% and 14.0% in controls, respectively. The frequency of minor allele was 43.7% in cases and 14.0% in controls. For rs7224837 A>G, CC, CT and TT genotype frequencies were 46.7%, 44.0%, and 9.3% in breast cancer cases versus 48.7%, 40.7% and 10.6% in controls, respectively. The frequency of minor allele was 31.3% in cases and 31.0% in controls. The estimated OR for association of AXIN2 s2240308 C>T, rs1133683 C>T, rs7224837 A>G polymorphisms are presented in Table 2. There was no significant distribution in genotype/allele of these variants between breast cancer cases and controls. Moreover, two dominant and recessive genetic models did not show a significant association between AXIN2 s2240308 C>T, rs1133683 C>T, rs7224837 A>G polymorphisms and breast cancer risk.

**Table 1:** Genotyping Features of AXIN2 Polymorphisms

SNP-ID	Position	Location	aa Change	Sequence Name	RE	Fragments
rs2240308	148 C>T	Exon 1	Pro to Ser	F: 5’-CCACGCCGATTGCTGAGAGG-3’	NsiI	C: 218, 24
				R: 5’-TTCCGCCTGGTGTTGGAAGAGACAT-3’		T: 242
rs1133683	1386 C>T	Exon 5	Pro to Pro	F: 5’-TGCGTAGGGAGCCGAATGTTG-3’	TaqI	C: 294
				R: 5’-GTGGTCCGGGGAGCGGGATC-3’		T: 274, 20
rs7224837	2406-1903 C>T	Intron 10	-	F: 5’-GATTACTCAACGCATCCCGGTC-3’	Hin1II	A: 211, 148
				F: 5’-CTCCCTGAGGCACAGTTAATAAG-3’		G: 359

**Table 2:** Distribution of the AXIN2 Polymorphisms in Breast Cancer Cases and Controls

Polymorphism	BC (n=150)	Controls (n=150)	Odds Ratio		
			OR	90% CI	P-Value
rs2240308 C>T					
Genotypes					
CC	72 (48.0)	84 (56.0)	Ref.		
CT	64 (42.7)	55 (36.7)	1.285	0.809-2.044	0.289
TT	14 (9.3)	11 (7.3)	1.301	0.570-2.966	0.532
Alleles					
C	208 (69.3)	223 (74.3)	Ref.		
T	92 (30.6)	77 (25.7)	1.281	0.897-1.830	0.174
Genetic Mode					
Dominant	86 (57.3)	139 (92.6)	0.106	0.053-0.213	0.001
Recessive	78 (52.0)	66 (44.0)	1.379	0.875-2.172	0.166
rs1133683 C>T					
Genotypes					
CC	46 (30.7)	49 (32.7)	Ref.		
CT	77 (51.3)	80 (53.3)	0.923	0.587-1.452	0.729
TT	27 (18.0)	21 (14.0)	1.348	0.724-2.510	0.346
Alleles					
C	169 (56.3)	178 (59.3)	Ref.		
T	131 (43.7)	122 (40.7)	1.131	0.818-1.564	0.457
Genetic Mode					
Dominant	123 (82.0)	129 (86.0)	0.742	0.398-1.381	0.346
Recessive	104 (69.3)	101 (67.3)	1.097	0.674-1.785	0.71
rs7224837 A>G					
Genotypes					
CC	70 (46.7)	73 (48.7)	Ref.		
CT	66 (44.0)	61 (40.7)	1.146	0.725-1.813	0.559
TT	14 (9.3)	16 (10.6)	0.862	0.405-1.836	0.701
Alleles					
C	206 (68.7)	207 (69.0)	Ref.		
T	94 (31.3)	93 (31.0)	1.016	0.719-1.435	0.93
Genetic Mode					
Dominant	136 (90.6)	134 (89.3)	1.16	0.545-2.470	0.701
Recessive	80 (53.3)	77 (51.3)	1.083	0.689-1.705	0.729

## Discussion

The association between AXIN2 and breast cancer has been demonstrated in limited populations and their results were somewhat inconclusive. To the best knowledge, this was the first study evaluated the association of AXIN2 polymorphisms with breast cancer in Iranian women. This study results revealed that AXIN2 rs2240308, rs7224837 and rs1133683 polymorphisms did not contribute to increased risk of breast cancer. Therefore, further studies in Iranian population should be conducted to explain the association between AXIN2 polymorphisms and breast cancer. Aristizabal-Pachon et al., for the first time, showed a significant association between AXIN2 rs151279728 and rs2240308 polymorphisms and breast cancer susceptibility in a Brazilian population, which indicating the role of Wnt/β-catenin pathway dysfunction in breast cancer tumorigenesis (Aristizabal-Pachon et al, 2015). In 2019, Dai et al., in a population of 415 n the Chinese Han breast cancer women and 528 healthy subjects evaluated the association of AXIN2 polymorphism with breast cancer. Their findings revealed that the rs11079571 and rs3923087 were associated with an increased risk of breast cancer, but not rs3923086. Moreover, they showed that the minor allele of rs3923087 polymorphism was associated with lymph node metastases and the ATA haplotype was correlated with an increased risk of breast cancer (Dai et al., 2019). Menezes et al., (2009) in a study assessed 75 families with nonsyndromic cleft lip with or without cleft palate (CL/P) and 93 control families without a history of cancer to find the role of *AXIN2* gene with cancer risk in families with CL/P. They showed that in those families the risk of cancer, especially colon cancer, was significantly increased. Alanazi et al., (2013) have performed a study to found association of 15 genetic variants located in 8 genes associated with Wnt signaling in Saudi Arabian women. Their results showed a significant association of genetic variants including *AXIN2 rs3923087, β-catenin rs4135385, DKK3 rs6485350, SFRP3 rs7775* and *TCF7L2 rs12255372* with breast cancer risk in the population. However, the study did not find a significant association of AXIN2 rs4791171, rs11079571 and rs3923086, β-catenin rs13072632, and SFRP3 rs288326 polymorphisms with breast cancer. Moreover, their results revealed that AXIN2 rs3923086 has a protective influence in breast cancer patients older than 43 years and AXIN2 rs3923087 heterozygous AG genotype was associated with reduced risk in both the age groups (≤43 or >43) as well as in the overall study. Wang et al., (2008) examined a number of SNPs in the *AXIN2 *gene including the four variants in this study and reported a significantly elevated risk with premenopausal breast cancers. In a Japanese population a SNP at codon 50 of the *AXIN2* gene was shown to have a significantly reduced risk of developing lung cancer while association with head and neck and colorectal cancer was not observed (Kanzaki et al, 2006).

Recently, Li et al., (2021) in a meta-analysis based on 72 studies with 22,087 cases and 18,846 controls evaluated the association of AXIN2 rs11079571, rs1133683 and rs35285779 polymorphisms with overall cancer risk. The meta-analysis results revealed that AXIN2 rs11079571, rs1133683 and rs35285779 polymorphisms were significantly associated with overall cancer risk. Gong et al., (2015) in a meta-analysis based on 8 studies with 1,559 cancer cases and 1503 controls evaluated the association of AXIN2 rs2240308 polymorphism with cancer risk. Their results revealed that this polymorphism was significantly associated with risk of cancer in overall. AXIN2 rs2240308 polymorphism was not associated with cancer risk in Turkish and Japanese populations. Their results showed that AXIN2 rs2240308 polymorphism was correlated with decreased risk of cancer by ethnicity. Yu et al., (2017) in a meta-analysis based on 14 studies with 2,215 cases and 2,481 controls found that AXIN2 rs2240308 polymorphism was not associated with cancer risk in overall. Similar with the previous meta-analysis, they showed that this genetic variant was associated with a decreased cancer risk in Asians.

In conclusion, this case-control study indicated that AXIN2 rs2240308, rs7224837 and rs1133683 polymorphisms did not contribute to increased risk of breast cancer. Further multicenter, prospective studies with larger sample size would have improved the accuracy of the present findings.

## Author Contribution Statement

Conceptualization: Soheila Sayad, Jamal Jafari-Nedooshan, Mahdieh Abdi-Gamsae. Data curation: Seyed Alireza Dastgheib, Hossein Neamatzadeh. Formal analysis: Seyed Alireza Dastgheib, Meraj Farbod. Investigation: Mahdieh Abdi-Gamsae, Hossein Neamatzadeh. Methodology: Seyed Alireza Dastgheib, Hossein Neamatzadeh, Fatemeh Asadian. Sampling: Jamal Jafari-Nedooshan, Meraj Farbod, Fatemeh Asadian. Resources: Mojgan Karimi-Zarchi, Mahdieh Abdi-Gamsae, Jamal Jafari-Nedooshan. Software: Soheila Sayad, Seyed Alireza Dastgheib, Hossein Neamatzadeh. Validation: Mojgan Karimi-Zarchi, Jamal Jafari-Nedooshan. Writing-original draft: Soheila Sayad, Mahdieh Abdi-Gamsae, Hossein Neamatzadeh. Writing-review and editing: all authors.

## Data Availability

The datasets generated during and/or analyzed during this study are the corresponding author on reasonable request.

## References

[B1] Alanazi MS, Parine NR, Shaik JP (2013). Association of single nucleotide polymorphisms in Wnt signaling pathway genes with breast cancer in Saudi patients. PLoS One.

[B2] Aristizabal-Pachon AF, Carvalho TI, Carrara HH, Andrade J, Takahashi CS (2015). AXIN2 polymorphisms, the β-catenin destruction complex expression profile and breast cancer susceptibility. Asian Pac J Cancer Prev.

[B3] Bahrami R, Shajari A, Aflatoonian M (2020). Association of REarranged during Transfection (RET) c 73 + 9277T > C and c 135G > a Polymorphisms with Susceptibility to Hirschsprung Disease: A Systematic Review and Meta-Analysis. Fetal Pediatr Pathol.

[B4] Bellanger M, Zeinomar N, Tehranifar P, Terry MB (2018). Are global breast cancer incidence and mortality patterns related to country-specific economic development and prevention strategies?. J Global Oncol.

[B5] Bianchini G, Balko JM, Mayer IA, Sanders ME, Gianni L (2016). Triple-negative breast cancer: Challenges and opportunities of a heterogeneous disease. Nat Rev Clinl Oncol.

[B6] Dai J, Gao H, Xue J, Lin W, Zheng L (2019). The association between AXIN2 gene polymorphisms and the risk of breast cancer in Chinese women. Genet Test Mol Biomarkers.

[B7] Esmaeili R, Mohammadi S, Jafarbeik-Iravani N (2021). Expression of SCUBE2 and BCL2 predicts favorable response in ERα positive breast cancer. Arch Iran Med.

[B8] Feng Y, Spezia M, Huang S (2018). Breast cancer development and progression: Risk factors, cancer stem cells, signaling pathways, genomics, and molecular pathogenesis. Genes Dis.

[B9] Forat-Yazdi M, Neamatzadeh H, Sheikhha MH, Zare-Shehneh M, Fattahi M (2015). BRCA1 and BRCA2 common mutations in iranian breast cancer patients: a meta analysis. Asian Pac J Cancer Prev.

[B10] Francies FZ, Hull R, Khanyile R, Dlamini Z (2020). Breast cancer in low-middle income countries: abnormality in splicing and lack of targeted treatment options. Am J Cancer Res.

[B11] Gajos-Michniewicz A, Czyz M (2020). Wnt signaling in melanoma. Int J Mol Sci.

[B12] Ghorbani F, Javadirad SM, Amirmahani F, Fatehi Z, Tavassoli M (2021). Associations of BCL2 CA-repeat polymorphism and breast cancer susceptibility in Isfahan province of Iran. Biochem Genet.

[B13] Gong J, Jiang Y, Hao N, Zhu B, Li Y (2015). Quantitative assessment of the association between AXIN2 rs2240308 polymorphism and cancer risk. Sci Rep.

[B14] Gooding AJ, Schiemann WP (2020). Epithelial-mesenchymal transition programs and cancer stem cell phenotypes: Mediators of breast cancer therapy resistance. Mol Cancer Res.

[B15] Guan R, Zhang X, Guo M (2020). Glioblastoma stem cells and Wnt signaling pathway: Molecular mechanisms and therapeutic targets. Chin Neurosurgical J.

[B16] Jarahzadeh MH, Asadian F, Farbod M (2021). Cancer and Coronavirus Disease (COVID-19): Comorbidity, Mechanical Ventilation, and Death Risk. J Gastrointestinal Cancer.

[B17] Kamali E, Tavassoli M, Hemmati S (2015). Association between the polymorphism of CA dinucleotide repeat in intron 1 of NFκB1 gene and risk of breast cancer. J Shahrekord Univ Med Sci.

[B18] Kanzaki H, Ouchida M, Hanafusa H (2006). Single nucleotide polymorphism of the AXIN2 gene is preferentially associated with human lung cancer risk in a Japanese population. Int J Mol Med.

[B19] Koni M, Pinnarò V, Brizzi MF (2020). The wnt signalling pathway: A tailored target in cancer. Int J Mol Sci.

[B20] Li X, Li Y, Liu G, Wu W (2021). New insights of the correlation between AXIN2 polymorphism and cancer risk and susceptibility: evidence from 72 studies. BMC Cancer.

[B21] Li N, Lim BWX, Thompson ER (2021). Investigation of monogenic causes of familial breast cancer: data from the BEACCON case-control study. NPJ Breast Cancer.

[B22] Liu Z, Wang P, Wold EA (2021). Small-molecule inhibitors targeting the canonical WNT signaling pathway for the treatment of cancer. J Med Chem.

[B23] De Medina P, Genovese S, Paillasse MR (2010). Auraptene is an inhibitor of cholesterol esterification and a modulator of estrogen receptors. Mol Pharmacol.

[B24] Menezes R, Marazita ML, Goldstein McHenry T (2009). AXIS inhibition protein 2, orofacial clefts and a family history of cancer. J Am Dent Assoc.

[B25] Moghimi M, Kargar S, Jafari MA (2018). Angiotensin converting enzyme insertion/deletion polymorphism is associated with breast cancer risk: A Meta-Analysis. Asian Pac J Cancer Prev.

[B26] Möller S, Mucci LA, Harris JR (2016). The heritability of breast cancer among women in the nordic twin study of cancer. Cancer Epidemiol Biomarkers Prev.

[B27] Motamedi S, Majidzadeh K, Mazaheri M (2012). Tamoxifen resistance and CYP2D6 copy numbers in breast cancer patients. Asian Pac J Cancer Prev.

[B28] Najminejad H, Farhadihosseinabadi B, Dabaghian M (2020). Key regulatory miRNAs and their interplay with mechanosensing and mechanotransduction signaling pathways in breast cancer progression. Mol Cancer Res.

[B29] Neamatzadeh H, Shiryazdi SM, Kalantar SM (2015). BRCA1 and BRCA2 mutations in Iranian breast cancer patients: A systematic review. J Res Med Sci.

[B30] Noroozi N, Dastgheib SA, Lookzadeh MH (2020). Association of axis inhibition protein 2 polymorphisms with non-syndromic cleft lip with or without cleft palate in Iranian children. Fetal Pediat Pathol.

[B31] Ren L, Chen H, Song J (2019). MiR-454-3p-mediated wnt/β-catenin signaling antagonists suppression promotes breast cancer metastasis. Theranostics.

[B32] Sheikhpour E, Noorbakhsh P, Foroughi E (2018). A survey on the role of interleukin-10 in breast cancer: A Narrative. Rep Biochem Mol Biol.

[B33] Sung H, Ferlay J, Siegel RL (2021). Global cancer statistics 2020: GLOBOCAN estimates of incidence and mortality worldwide for 36 cancers in 185 countries. CA Cancer J Clin.

[B34] Tavakoli-Koopaei R, Javadi-Zarnaghi F, Aghahosseini M, Tavassoli M, Rasaee MJ (2020). Performance of a template enhanced hybridization process in biological media for the detection of a breast cancer biomarker. Anal Methods.

[B35] Wang X, Goode EL, Fredericksen ZS (2008). Association of genetic variation in genes implicated in the β-catenin destruction complex with risk of breast cancer. Cancer Epidemiol Biomarkers Prev.

[B36] Xu X, Zhang M, Xu F, Jiang S (2020). Wnt signaling in breast cancer: biological mechanisms, challenges and opportunities. Mol Cancer.

[B37] Yu Y, Tao Y, Liu L (2017). New concept of the Axin2 rs2240308 polymorphism and cancer risk: An updated meta-analysis. Neoplasma.

